# Preliminary study on the function of the *POLD1* (*CDC2*) EXON2 c.56G>A mutation

**DOI:** 10.1002/mgg3.1280

**Published:** 2020-05-20

**Authors:** Jing Liu, Yu Liu, Jingxuan Fu, Chengeng Liu, Tingting Yang, Xiaomin Zhang, Min Cao, Peichang Wang

**Affiliations:** ^1^ Department of Clinical Laboratory Xuanwu Hospital Capital Medical University Beijing People's Republic of China; ^2^ Department of Clinical Laboratory The Hospital of Shunyi District Beijing Beijing People's Republic of China

**Keywords:** DNA polymerase δ, Fanconi anemia, *POLD1* mutation

## Abstract

**Background:**

Fanconi anemia (FA) is a rare recessive disease characterized by DNA damage repair deficiency, and DNA polymerase δ (whose catalytic subunit is encoded by *POLD1*, also known as *CDC2*) is closely related to DNA damage repair. Our previous study identified a novel *POLD1* missense mutation c.56G>A (p. Arg19>His) in FA family members. However, the function of the *POLD1* missense mutation is currently unknown. This study aimed to uncover the biological function of the *POLD1* missense mutation.

**Methods:**

Stable cell lines overexpressing wild‐type *POLD1* or mutant *POLD1* (c.56G>A, p.Arg19His) were constructed by lentivirus infection. Cell growth curve analysis, cell cycle analysis, and a comet assay were used to analyze the function of the *POLD1* mutation.

**Results:**

The growth and proliferative ability of the cells with *POLD1* mutation was decreased significantly compared with those of the wild‐type cells (Student's *t* test, *p* < .05). The percentage of cells in the G0/G1 phase increased, and the percentage of cells in the S phase decreased significantly when *POLD1* was mutated (Student's *t* test, *p* < .05). Moreover, the Olive tail moment value of the cells with the *POLD1* mutation was significantly higher than that of the cells with wild‐type *POLD1* after H_2_O_2_ treatment.

**Conclusions:**

The *POLD1* mutation inhibited cell proliferation, slowed cell cycle progression, and reduced DNA damage repair.

## Graphical abstract

A novel POLD1 missense mutation (c.56G>A, p.Arg19>His) inhibited cell proliferation, slowed cell cycle progression and reduced DNA damage repair.

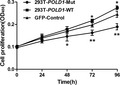

## 1 INTRODUCTION

Fanconi anemia (FA) is a rare autosomal or X chromosome‐linked recessive hereditary disease characterized by DNA damage repair deficiency and high cancer susceptibility. DNA polymerase δ is one of the important polymerases participating in eukaryotic DNA synthesis, damage, and repair; cell cycle control; and other processes, and abnormalities of this polymerase are closely related to the occurrence and development of cancer (Bellido et al., 2016; Buchanan, Rosty, Clendenning, Spurdle, & Win, 2014; Jansen et al., 2016; Lek et al., 2016; Palles et al., 2013). Hence, we screened mutated sites in *POLD1* (OMIM accession number: 174761), also known as *CDC2*, which encodes the DNA polymerase δ catalytic subunit in FA family members. The 15 family members of FA family constellation were enrolled in the study. DNA polymerase gene family members including *POLA*, *POLB*, *POLD1*, *POLD2*, *POLE*, and *POLG* of FA proband were analyzed by gene sequencing to screen meaningful mutation sites, and the DNA sequence of the family members was analyzed by direct sequencing in plus sense mutation region of the proband. As a result, the *POLD1* EXON2 c.56G>A (p.Arg19His) mutation was found in three FA family members (Liu & Wang, 2016). The *POLD1* c.56G>A (p.Arg19His) mutation is localized in the nuclear localization sequence of DNA polymerase δ. However, the underlying function of the *POLD1* mutation is currently unknown. To analyze the significance of the *POLD1* mutation, growth, proliferation, cell cycling, and DNA damage repair were analyzed in HEK293T‐*POLD1*‐WT and HEK293T‐*POLD1*‐Mut cells.

## 2 MATERIALS AND METHODS

### 2.1 Ethical compliance

This article does not contain any studies with human participants or animals performed by any of the authors. The authors are accountable for all aspects of the work.

### 2.2 Cell lines

*POLD1* (GenBank, NG_033800.1, NM_001308632.1) was cloned to construct HEK293T‐*POLD1*‐WT (293T‐*POLD1*‐WT) stable cell line, mutant *POLD1* (c.56G>A, p.Arg19His) was cloned to construct HEK293T‐*POLD1*‐Mut (293T‐*POLD1*‐Mut) stable cell line, and these two cell lines and HEK293T‐GFP‐PURO (GFP‐Control) stable cell line were constructed by Hanbio Technology Co., Ltd. HEK293T (Control) cell line was purchased from National Infrastructure of Cell Line Resource.

### 2.3 Western blot analysis

Western blotting was performed according to the protocol of a previous study (Wang & Wang, 2012). Cells were lysed using RIPA buffer (Solarbio) with a protease inhibitor mixture (Solarbio), and the cell lysates were then centrifuged for 30 min at 12,000 *g* at 4°C. The supernatants were collected. After the protein concentration was determined by BCA protein assay kit (Invitrogen), total protein (30–50 μg) was loaded for separation by sodium dodecyl sulfate‐polyacrylamide gel electrophoresis (SDS‐PAGE) and then transferred to PVDF membranes (Millipore). Following blocking in 5% nonfat milk for 1 hr at room temperature, the membranes were incubated with a primary antibody for POLD1 (Abcam, ab186407) and β‐actin (Abcam, ab8226) overnight at 4°C. Then, the membranes were incubated with a HRP‐conjugated secondary antibody (ZSGB‐BIO) for 1 hr at room temperature after washing three times with TBST. Following washing, protein bands were visualized using a chemiluminescent substrate (Millipore) according to the manufacturer's instructions.

### 2.4 Growth curve assay

Growth curves were generated using a Cell Counting Kit‐8 (CCK‐8) assay (Beyotime) performed according to the manufacturer's protocol. Briefly, cells were prepared in a 96‐well plate at a density of 3 × 10^3^ per well in four replicates. Then, 10 μl of CCK‐8 reagent was added to each well of the plate and incubated for 3 hr at 37°C. The absorbance at 450 nm was measured using a microplate reader. The absorbance was detected at 0, 24, 48, 72, and 96 hr after plating.

### 2.5 Cell cycle analysis

Cells were digested with 0.25% trypsin (Invitrogen). The suspended cells were collected by centrifugation at 188 g for 5 min and then washed twice with cold PBS. Then, the cells were fixed with 70% ethanol overnight at 4°C. After washing the cells with cold PBS twice, RNase A (Invitrogen) was added and incubated at 37°C for 30 min for RNA digestion. Then, 10 µl of propidium iodide (PI) (Millipore) was added for 15 min at room temperature and incubated protected from light for staining. A BD FACS flow cytometer was used to analyze the DNA content.

### 2.6 Comet assay

A comet assay was performed as previously described (Wang & Wang, 2012). Briefly, cells were resuspended at a concentration of 1 × 10^5^ cells/ml, and then 5 ml of H_2_O_2_ (100 μM) was added. The cells were incubated with H_2_O_2_ for 10 min at 4°C in the dark. Following washing with PBS, the cells were cultured for 1 hr at 37°C and collected for the comet assay using Comet Assay kit (Trevigen). Slides were stained with ethidium bromide (5 μg/ml) for 20 min. Images were acquired using a fluorescence microscope with an excitation filter at 496 nm and a blocking filter at 522 nm. A Leica image analysis system (Q550CW) was used to analyze the results of the comet assay. One hundred cells were selected randomly, and the Olive tail moment (OTM) was measured to indicate the status of DNA fracture and damage.

### 2.7 Statistical analysis

Student's *t* test was used for statistical analyses. Differences were considered significant at *p* < .05.

## 3 RESULTS

### 3.1 Morphology of cells with the *POLD1* c.56G>A (p.Arg19His) mutation

**FIGURE 1:**
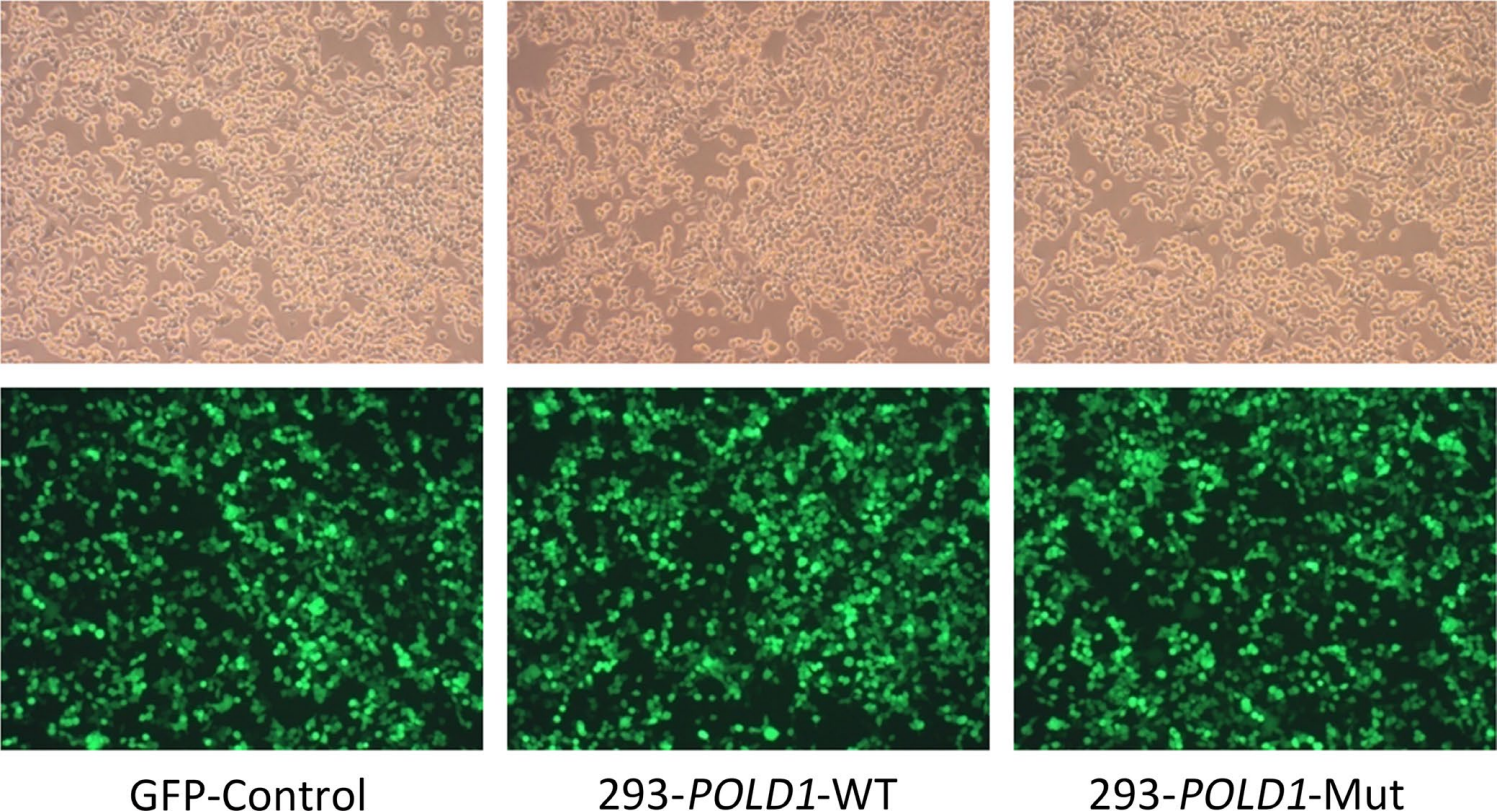
Cell morphology of GFP‐control, 293T‐*POLD1*‐WT, and 293T‐*POLD1*‐Mut cells. These cells morphology analyzed using fluorescence microscopy and light microscopy. Cells were seeded in 6‐well plates. After incubating for 48 hr, images were acquired by light microscopy (top) and fluorescence microscopy (bottom)

The morphologies of GFP‐Control, 293T‐*POLD1*‐WT, and 293T‐*POLD1*‐Mut cells were observed using fluorescence microscopy and light microscopy. As shown in Figure 1, there were no differences among the GFP‐Control, 293T‐*POLD1*‐WT, and 293T‐*POLD1*‐Mut cells in size or morphology. These results indicated that cell morphology was not altered by the *POLD1* c.56G>A (p.Arg19His) mutation.

### 3.2 Expression levels of *POLD1* in 293T‐*POLD1*‐WT and 293T‐*POLD1*‐Mut cells

**FIGURE 2:**
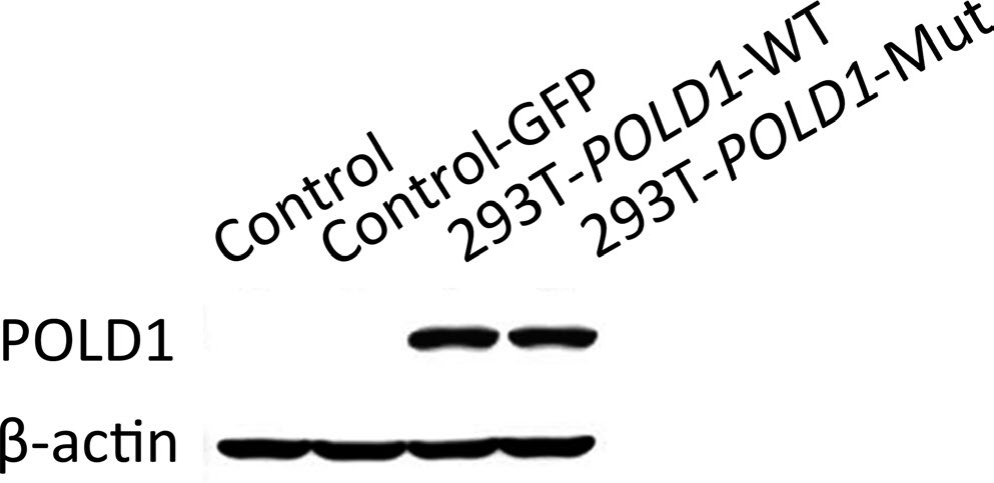
The expression levels of *POLD1* in 293T‐*POLD1*‐Mut cells. Cells were collected and lysed with RIPA buffer. After the protein concentration was determined, the lysates were subjected to Western blotting and probed with anti‐POLD1 and anti‐β‐actin antibodies

The expression levels of *POLD1* were measured in Control, GFP‐Control, 293T‐*POLD1*‐WT, and 293T‐*POLD1*‐Mut cells using Western blotting. Compared with the Control and GFP‐Control cell lines, the 293T‐*POLD1*‐WT and 293T‐*POLD1*‐Mut stable cell lines showed *POLD1* overexpression (Figure 2), indicating that the 293T‐*POLD1*‐Mut and 293T‐*POLD1*‐WT cell lines were obtained successfully and the expression of *POLD1* was not affected by the *POLD1* c.56G>A (p.Arg19His) mutation.

### 3.3 Cell growth and proliferation of 293T‐*POLD1*‐WT and 293T‐*POLD1*‐Mut cells

**FIGURE 3:**
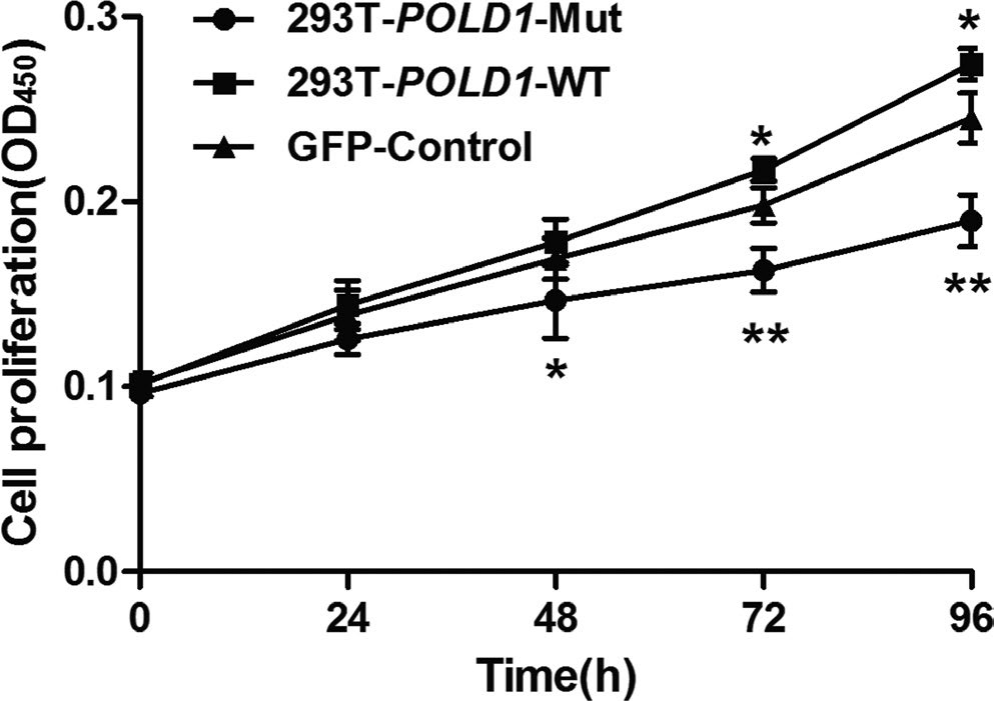
Cell growth curves of 293T‐*POLD1*‐WT and 293T‐*POLD1*‐R19H mutant cells. Cells were seeded in 96‐well plates. The cell growth curves of 293T‐*POLD1*‐WT and 293T‐*POLD1*‐Mut mutant cells were determined by a CCK‐8 assay. Data are shown as the mean ± standard deviation (*SD*) from four independent experiments. The data were compared by two‐way ANOVA, with four independent experiments in each group; *p < .05, **p < .01, ***p < .005

The cell growth and proliferation of 293T‐*POLD1*‐Mut cells were analyzed using a CCK‐8 assay. Compared with GFP‐Control cells, the growth and proliferation of 293T‐*POLD1*‐WT cells were increased, whereas the growth and proliferation of 293T‐*POLD1*‐Mut cells were decreased significantly (Figure 3). These results indicated that the *POLD1* c.56G>A (p.Arg19His) mutation inhibited cell growth and proliferation, which could be attributed to an alteration in POLD1 function caused by the mutation.

### 3.4 Cell cycle analysis of 293T‐*POLD1*‐WT and 293T‐*POLD1*‐Mut cells

**FIGURE 4:**
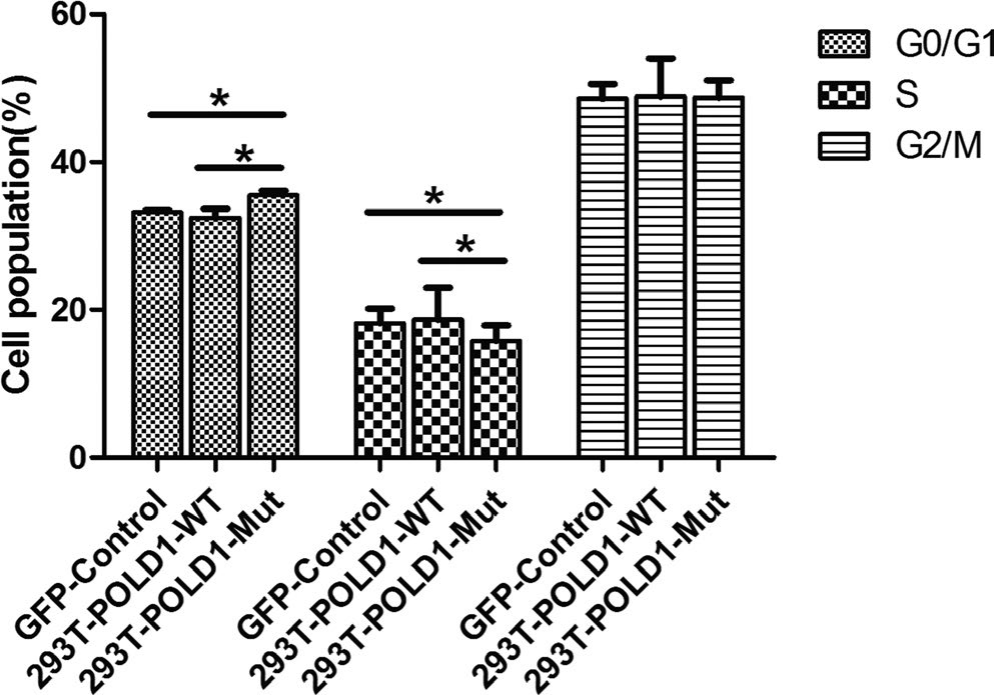
Cell cycle analysis of 293T‐*POLD1*‐Mut, 293T‐*POLD1*‐WT, and GFP‐control cells. Cells were stained with PI and then analyzed by flow cytometry. Data are shown as the mean ± standard deviation (*SD*) from four independent experiments. The data were compared by one‐way ANOVA, with four independent experiments in each group; *p < .05, **p < .01, ***p < .005

The cell cycles of 293T‐*POLD1*‐Mut and 293T‐*POLD1*‐WT cells were analyzed. As shown in Figure 4, the percentage of cells in the G0/G1 phase in the 293T‐*POLD1*‐Mut group was significantly higher than that in the 293T‐*POLD1*‐WT and GFP‐control cell groups (Student's *t* test, *p* < .05). In addition, the percentage of cells in the S phase in the 293T‐*POLD1*‐Mut group was significantly lower than that in the 293T‐*POLD1*‐WT and GFP‐control cell groups (Student's *t* test, *p* < .05). These results suggested that the mutation slowed the cell cycle, which could contribute to the decreases in growth and proliferation induced by the mutation. The slowdown of the cell cycle caused by the mutation could be attributed to a decline in DNA replication.

### 3.5 DNA damage repair abilities of 293T‐*POLD1*‐Mut and 293T‐*POLD1*‐WT cells

**FIGURE 5:**
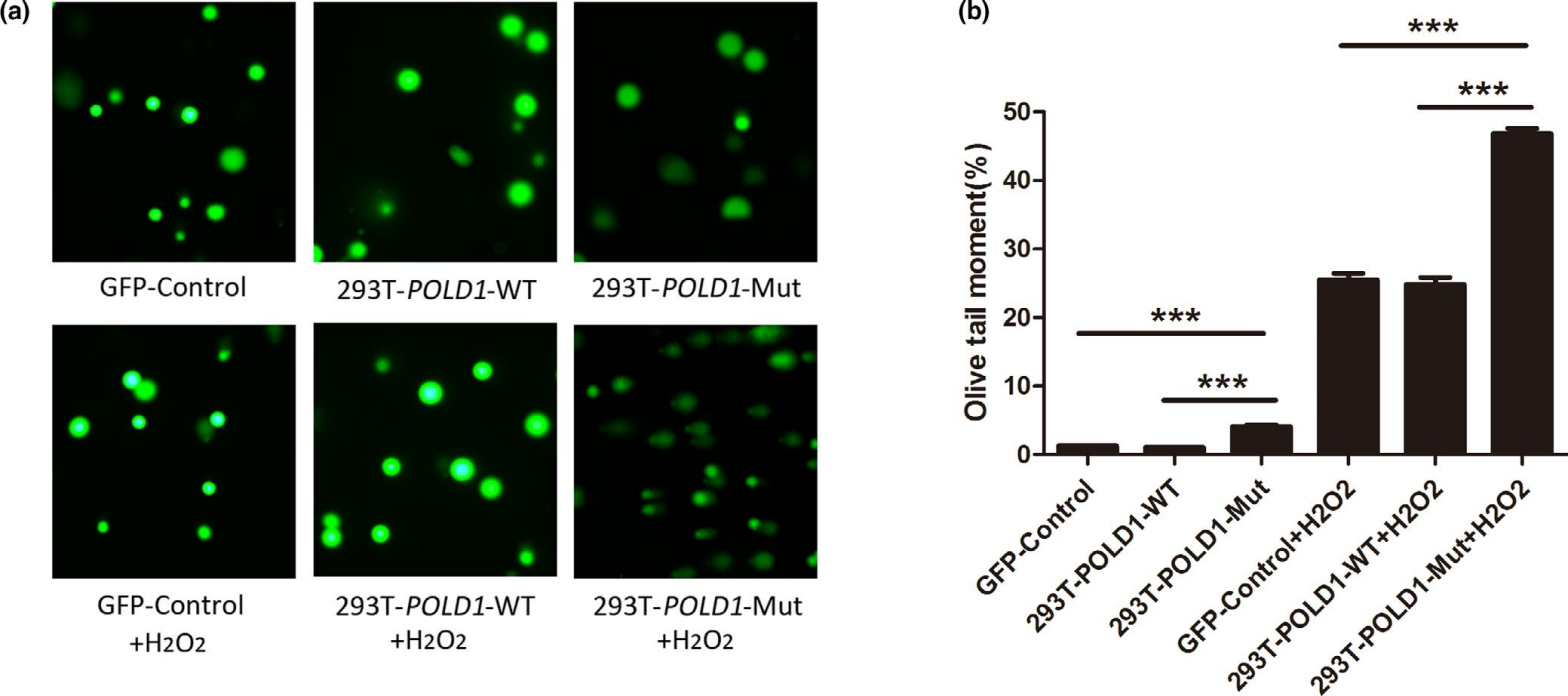
Comet assay results for 293T‐*POLD1*‐Mut, 293T‐*POLD1*‐WT, and GFP‐control cells. (a) Comet assay for 293T‐*POLD1*‐Mut, 293T‐*POLD1*‐WT, and GFP‐control cells. (b) The olive tail moment was determined by CAPS software (*n* = 50). *p < .05, **p < .01, ***p < .005

293T‐*POLD1*‐Mut, 293T‐*POLD1*‐WT, and GFP‐Control cells were collected and incubated with H_2_O_2_ for 5 min at 4°C. Then, the cells were cultured for 1 hr. The DNA damage repair abilities of these cells were analyzed with a comet assay. As shown in Figure 5, the length of the comet tail (Figure 5a) and OTM value (Figure 5b) of the 293T‐*POLD1*‐Mut cells were increased significantly compared with those of the 293T‐*POLD1*‐WT cells and GFP‐Control cells at the 100 μmol/l doses of H_2_O_2_ (*p* < .05). These results suggested that the *POLD1* c.56G>A (p.Arg19His) mutation reduced DNA damage repair, even though mutated *POLD1* was overexpressed.

## 4 DISCUSSION

Fanconi anemia is a rare autosomal or X chromosome‐linked recessive hereditary disease that generally has 5‐ to 10‐year morbidity (Mamrak, Shimamura, & Howlett, 2017). In addition to FA patients having high rates of myelodysplastic syndrome and acute myelocytic leukemia (AML), the cumulative incidence of solid tumors in FA patients is 76% (Reina‐Castillon et al., 2017). FA has a variety of clinical manifestations, such as slight anemia, serious aplastic anemia, and even AML. These variations indicate that large individual differences exist. In addition to evaluations of clinical manifestations and routine inspections that are used for diagnosis, other methods should be utilized to assist in diagnosis, such as gene analysis.

*POLD1* is the coding gene for the DNA polymerase δ catalytic subunit. As the most important replicase, DNA polymerase δ not only participates in DNA replication but also plays an important role in the DNA damage repair process in several forms, which is of great significance in maintaining the cell cycle and guaranteeing genomic structural integrity and genetic stability (Bartels, Stodola, Burgers, & Barton, 2017; Lee et al., 2012; Nicolas, Golemis, & Arora, 2016). DNA pol δ belongs to the DNA polymerase B family (Zhou, Meng, Zhang, Lee, & Lee, 2012). In 1976, So.AG discovered that DNA pol δ has 3′‐5′ exonuclease activity in eukaryotic cells. This polymerase has four subunits (p125, p68, p50, and p12), and the coding gene for the catalytic subunit p125 (*POLD1*) is in the q13.3–q13.4 area of chromosome 19. The cDNA sequence of *POLD1* is 3,443 bp, and it has 27 exons and 26 introns. p125 is composed of 1,107 amino acid residues.

DNA pol δ participates in DNA mismatch repair, translesion synthesis, base excision repair, nucleotide excision repair, etc. The mismatch repair system is of great importance in maintaining the stability of the genome. A deficiency in mismatch repair may lead to mutant genes and enhance cancer susceptibility (Lee et al., 2014), so abnormalities in DNA polymerase δ have close relationships with the occurrence and development of cancer. Some research has demonstrated that Werner syndrome and Bloom's syndrome (BLM) are related to DNA pol δ. Kamatlh‐Loeb discovered that the Werner syndrome‐related gene WRN might combine with the p66 subunit of DNA pol δ and further mediate DNA replication and damage repair. A fracture or an abnormality in the WRN‐DNA pol δ complex results in insufficient DNA synthesis (Zhang et al., 2012). A recent study showed that BLM gene could interact with the DNA pol δ p12 subunit to activate the DNA helicase activity of the BLM gene. In contrast, the BLM gene could activate the DNA pol δ activity of displacement through the p12 subunit (Patel, Misenko, Her, & Bunting, 2017).

In a preliminary study, we detected the *POLD1* c.56G>A (p.Arg19His) mutation in the family of a diagnosed FA patient. To analyze the biological effect of the *POLD1* c.56G>A (p.Arg19His) mutation, control cells stably transfected with lentiviral vectors containing *POLD1* with or without the mutation site were prepared. The expression levels of *POLD1* in 293T‐*POLD1*‐Mut and 293T‐*POLD1*‐WT cells were higher than those in control and GFP‐Control cells, suggesting that the cell lines were obtained successfully and the expression of *POLD1* was not altered by the mutation.

Cell growth and proliferation were analyzed with a CCK‐8 assay. The results showed that growth and proliferation were increased in 293T‐*POLD1*‐WT cells, whereas growth and proliferation were significantly decreased in 293T‐*POLD1*‐Mut cells compared with GFP‐control cells. These results indicated that the *POLD1* c.56G>A (p.Arg19His) mutation decreased cell growth and proliferation, which could be attributed to alteration of POLD1 function by the mutation.

The cell cycles of 293T‐*POLD1*‐Mut and 293T‐*POLD1*‐WT cells were also analyzed. The results showed that the percentage of cells in the G0/G1 phase in the 293T‐*POLD1*‐Mut group was significantly higher than that in the 293T‐*POLD1*‐WT and GFP‐control cell groups. In addition, the percentage of cells in the S phase in the 293T‐*POLD1*‐Mut group was significantly lower than that in the 293T‐*POLD1*‐WT and GFP‐control cell groups. These results suggested that the mutation slowed the cell cycle, which could contribute to the decreases in growth and proliferation induced by the mutation. The slowdown of the cell cycle caused by the mutation could be attributed to a decline in the DNA synthetase activity of DNA pol δ harboring the mutation.

The comet assay is also called single‐cell gel electrophoresis, which can intuitively reflect the DNA single‐strand breakage level. In this study, H_2_O_2_‐treated cells were cultured for 1 hr, and a comet assay was performed. The results showed that the OTM value of 293T‐*POLD1*‐Mut cells was significantly higher than that of 293T‐*POLD1*‐WT and GFP‐control cells. These results strongly suggested that the *POLD1* c.56G>A (p.Arg19His) mutation reduced DNA damage repair, which could contribute to the declines in DNA damage detection and DNA synthesis induced by the mutation. In addition, these results explained the slowdown of the cell cycle and the decreases in cell growth and proliferation caused by the *POLD1* c.56G>A (p.Arg19His) mutation.

## CONFLICT OF INTEREST

The authors declare no conflict of interest.

## AUTHOR CONTRIBUTIONS

Jing Liu and Yu Liu designed and performed experiments, and analyzed data. Jing Liu wrote the study; Jingxuan Fu, Chengeng Liu, and Tingting Yang analyzed data; Xiaomin Zhang and Min Cao performed some experiments; Peichang Wang initiated the study, organized, designed, and wrote the study.

## Data Availability

The authors declare that the data supporting the findings of this study are available within the article.
