# Correction to “Preliminary study on the function of the *POLD1* ( *CDC2* ) EXON2 c.56G>A mutation”

**DOI:** 10.1002/mgg3.2230

**Published:** 2023-06-27

Liu, J, Liu, Y, Fu, J, et al. Preliminary study on the function of the *POLD1* (*CDC2*) EXON2 c.56G>A mutation. *Mol Genet Genomic Med*. 2020; 8:e1280. https://doi.org/10.1002/mgg3.1280

In the originally published article, in Figure 1, an incorrect set of images for GFP‐Control and 293‐POLD1‐Mut were presented. The correct images are provided below.

**FIGURE 1:**
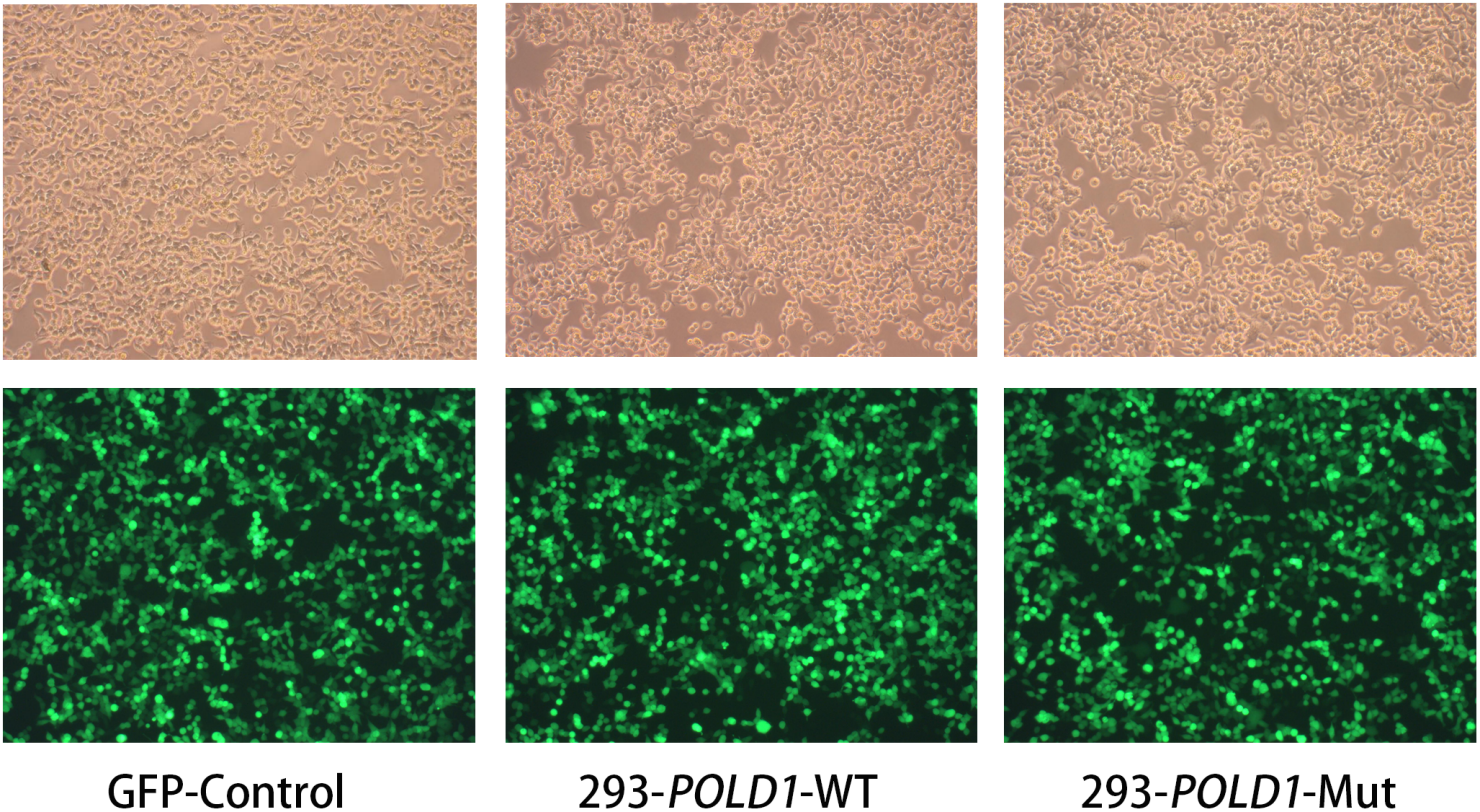
Cell morphology of GFP‐control, 293T‐*POLD1*‐WT, and 293T‐*POLD1*‐Mut cells. These cells morphology analyzed using fluorescence microscopy and light microscopy. Cells were seeded in six‐well plates. After incubating for 48 h, images were acquired by light microscopy (top) and fluorescence microscopy (bottom).

We apologize for this error.

